# Antibody Secreting Cell Responses following Vaccination with Bivalent Oral Cholera Vaccine among Haitian Adults

**DOI:** 10.1371/journal.pntd.0004753

**Published:** 2016-06-16

**Authors:** Wilfredo R. Matias, Brie Falkard, Richelle C. Charles, Leslie M. Mayo-Smith, Jessica E. Teng, Peng Xu, Pavol Kováč, Edward T. Ryan, Firdausi Qadri, Molly F. Franke, Louise C. Ivers, Jason B. Harris

**Affiliations:** 1 Department of Global Health & Social Medicine, Harvard Medical School, Boston, Massachusetts, United States of America; 2 Partners In Health, Boston, Massachusetts, United States of America; 3 Division of Infectious Diseases, Massachusetts General Hospital, Boston, Massachusetts, United States of America; 4 Harvard Medical School, Boston, Massachusetts, United States of America; 5 Division of Global Health Equity, Brigham & Women’s Hospital, Boston, Massachusetts, United States of America; 6 NIDDK, LBC, National Institutes of Health, Bethesda, Maryland, United States of America; 7 International Centre for Diarrhoeal Disease Research Bangladesh (icddr,b), Dhaka, Bangladesh; 8 Department of Pediatrics, Harvard Medical School, Boston, Massachusetts, United States of America; University of California San Diego School of Medicine, UNITED STATES

## Abstract

**Background:**

The bivalent whole-cell (BivWC) oral cholera vaccine (Shanchol) is effective in preventing cholera. However, evaluations of immune responses following vaccination with BivWC have been limited. To determine whether BivWC induces significant mucosal immune responses, we measured *V*. *cholerae* O1 antigen-specific antibody secreting cell (ASC) responses following vaccination.

**Methodology/Principal Findings:**

We enrolled 24 Haitian adults in this study, and administered doses of oral BivWC vaccine 14 days apart (day 0 and day 14). We drew blood at baseline, and 7 days following each vaccine dose (day 7 and 21). Peripheral blood mononuclear cells (PBMCs) were isolated, and ASCs were enumerated using an ELISPOT assay. Significant increases in Ogawa (6.9 cells per million PBMCs) and Inaba (9.5 cells per million PBMCs) OSP-specific IgA ASCs were detected 7 days following the first dose (P < 0.001), but not the second dose. The magnitude of *V*. *cholerae*-specific ASC responses did not appear to be associated with recent exposure to cholera. ASC responses measured against the whole lipolysaccharide (LPS) antigen and the OSP moiety of LPS were equivalent, suggesting that all or nearly all of the LPS response targets the OSP moiety.

**Conclusions/Significance:**

Immunization with the BivWC oral cholera vaccine induced ASC responses among a cohort of healthy adults in Haiti after a single dose. The second dose of vaccine resulted in minimal ASC responses over baseline, suggesting that the current dosing schedule may not be optimal for boosting mucosal immune responses to *V*. *cholerae* antigens for adults in a cholera-endemic area.

## Author Summary

The bivalent whole-cell (BivWC) oral cholera vaccine (Shanchol) is effective in preventing cholera. Despite its increasing use as part of comprehensive cholera prevention and control efforts, evaluations of immune responses following vaccination with BivWC have been limited. In this study, we measured the development of cholera-specific antibody secreting cells, markers of mucosal immunity, following vaccination with BivWC among a population of adults in Haiti, where cholera is now endemic. BivWC induced development of robust immune responses following the first dose of vaccine, but similar ASC responses were not detected following the second dose, suggesting that the currently recommended 14-day interval between doses may not be optimal for boosting mucosal immune responses among adults in cholera endemic regions. These findings suggest that additional evaluation of the optimal dosing schedule for oral cholera vaccines is warranted with the goal of improving long-term immunity.

## Introduction

Cholera, a diarrheal disease with epidemic potential caused by the bacterium *Vibrio cholerae*, remains a significant cause of morbidity and mortality, accounting for approximately 1.4 to 4.3 million cases of acute diarrhea and 28,000 to 142,000 deaths annually.[1] *V*. *cholerae* serogroup O1 is the current predominant cause of cholera, while serogroup O139 was a major cause of cholera in the 1990s and early 2000s.[2] The O1 serogroup is further classified into Ogawa and Inaba serotypes.[3] These differ by the presence of a 2-O-methyl group present in the terminal carbohydrate of the O-specific polysaccharide (OSP) in the lipopolysaccharide (LPS) of the Ogawa serotype.[4–6] Protection against cholera is serogroup specific, and serogroup specificity is defined by the OSP moiety of LPS.[7–9]

Recent years have seen the frequency and severity of cholera outbreaks increase worldwide.[10] For example, the cholera epidemic that began in Haiti in October 2010 has accounted for 750,752 reported cases of acute diarrhea and 9,031 deaths,[11] making it the largest outbreak of cholera in recent history. While the development of robust water and sanitation infrastructure is essential in regions affected by cholera, vaccination offers another important and complementary tool for cholera prevention and control.[12]

There are currently three World Health Organization (WHO) pre-qualified, commercially available oral cholera vaccines (OCV): Dukoral (Crucell, Sweden), which consists of whole-cell killed *V*. *cholerae* O1 Inaba and Ogawa and recombinant cholera toxin B subunit (WC-rBS); and Shanchol (Shantha Biotechnics, India) and Euvichol (EuBiologics, Korea), which are similar bivalent whole-cell (BivWC) vaccines containing multiple biotypes of *V*. *cholerae* O1 and O139 which lack the cholera toxin B subunit (CtxB) found in Dukoral. Following Shanchol’s prequalification by the WHO in 2011, multiple programs in diverse settings including Haiti have demonstrated the feasibility and efficacy of integrating vaccination with BivWC into comprehensive cholera control strategies.[13,14] In 2013, the WHO created an OCV stockpile to respond to cholera outbreaks worldwide.[15]

Despite the rapidly growing use of oral cholera vaccination with BivWC, there remain significant gaps in our understanding of immune responses following vaccination with BivWC. Since vaccine immunogenicity is a surrogate for protection, such studies are especially important in settings where large scale evaluations of direct effectiveness are not practical, such as in comparing vaccine formulations, dosing schedules, and in estimating vaccine efficacy across specific populations. Trials to assess safety and immunogenicity have demonstrated development of significant vibriocidal antibody and OSP-specific plasma immunoglobulin A (IgA) responses among adults and children following vaccination[16–20], including comparable vibriocidal antibody responses following a 14-day versus a 28-day dosing interval in a cholera-endemic area of Kolkata, India.[18] Previously, our group demonstrated significant vibriocidal responses and OSP-specific IgA responses following vaccination with BivWC among adults, children, and HIV-infected adults in Haiti.[21,22] However, compared to studies characterizing immune responses following natural cholera infection[23–28] and vaccination with WC-rBS[29–33], studies evaluating immune responses following vaccination with BivWC are limited.

Among evaluations of immunogenicity, the detection of antibody secreting cells (ASCs) in blood is a standard measure of the mucosal immune response. Following stimulation in the gastrointestinal tract by live *V*. *cholera*e or by OCVs, these cells transiently migrate into the systemic circulation where they can be detected by standard Enzyme-Linked ImmunoSpot (ELISPOT) assays.[34] This migration peaks approximately at day 7 following primary infection or vaccination. Many of these circulating ASCs express gut-homing markers and return to mucosal tissue.[35] Detection of LPS-specific ASCs in the peripheral blood correlates with the development of *V*. *cholerae* antigen-specific antibodies in the small intestinal lamina propria up to 6 months following infection.[36] As such, ASCs provide a critical and early window into subsequent immunologic memory at the mucosal surface.

To determine whether vaccination with BivWC induces significant mucosal immune responses, we measured levels of ASC responses against *V*. *cholerae* LPS and OSP at multiple time points following vaccination with BivWC among healthy participants in Haiti.

## Methods

### Study Population and Study Design

Healthy adults, ages 18–60 years old attending the Saint Nicolas Hospital outpatient clinic in Saint Marc, Haiti were invited to participate in May 2015, and were deemed eligible if they lived in the catchment area. Individuals with an active gastrointestinal disorder, pregnancy (as determined by urine pregnancy test), those that had previously received OCV, and those unable to give informed consent were excluded from the study. The BivWC vaccine was stored at 2°C–8°C prior to administration. Two doses of vaccine were given 14 days apart, in accordance with the package insert. Venous blood from each participant was obtained prior to immunization and 7 days after each dose of vaccine (on days 0, 7, and 21). A stool specimen was collected on day 7 to test for enteric pathogens, and a questionnaire on social and nutritional factors was conducted on day 14. The participants enrolled in this study were part of a larger ongoing study to measure long-term immune responses among adult cholera vaccine recipients.

### Ethics Statement

Written informed consent was obtained from all potential adult participants prior to enrolling in the study. The study protocol was reviewed and approved by the institutional review board of Partners HealthCare in Boston, Massachusetts, and the Haitian National Bioethics Committee in Port-au-Prince, Haiti.

### Sample Collection and Isolation of PBMCs

We collected venous blood from each participant in Vacutainer blood collection tubes (Becton Dickinson), containing sodium heparin anticoagulant. Samples were obtained at day 0 (before vaccination), day 7 (7 days after the first dose of vaccine), and day 21 (1 week after the second dose of vaccine). Following sample collection, blood was diluted in PBS (pH 7.4) and processed for separation of plasma and peripheral blood mononuclear cells (PBMCs). PBMCs were prepared by differential centrifugation in Leucosep tubes (Greiner Bio-One Ltd) filled with Ficoll-Isopaque (Pharmacia, Piscataway, NJ), and after two subsequent PBS washes, resuspended at a concentration of 5 x 10^6^ cells/mL in RPMI complete medium (Gibco, Carlsbad, CA) supplemented with 10% heat-inactivated fetal bovine serum (FBS) (HyClone, Logan, UT) and 1% Penicillin-Streptamycin (Sigma-Aldrich). These cells were used immediately as described below for measuring ASC responses. Plasma was shipped in a liquid nitrogen dry vapor unit and stored at -80°C prior to analysis in Boston.

### Antibody Responses in Plasma

Vibriocidal antibody responses in plasma were measured using a standard protocol described previously.[24] Briefly, *V*. *cholerae* O1 El Tor Ogawa (X25049) and Inaba (T19479) were used as the target organism. The vibriocidal titer was defined as the reciprocal of the highest plasma dilutions resulting in a greater than 50% optical density reduction associated with *V*. *cholerae* O1 growth when compared to control wells without plasma. An increase of titer by 4-fold was considered to represent seroconversion, by convention. Control plasma, a pool of day 7 plasma from culture-confirmed *V*. *cholerae* infected individuals in Dhaka, Bangladesh, was used to monitor intra-assay variability between plates.

OSP was purified and conjugated to bovine serum albumin (BSA) as previously described;[37] source strains of OSP Inaba and Ogawa were *V*. *cholerae* O1 El Tor PIC018 and PIC058, respectively.[38] Anti-OSP IgA responses in plasma were measured using a previously described ELISA protocol.[24] In brief, 96-well polystyrene plates (Nunc, low affinity plates) were coated with *V*. *cholerae* O1 Ogawa OSP conjugated to BSA, or Inaba OSP conjugated to BSA (1 μg/mL), dissolved in 50mM carbonate buffer (pH 9.6). We added 100 μL of plasma diluted 1:25 in 0.1% BSA in PBS-0.05% Tween. Plates were incubated at 37°C for 60 minutes while shaking (100 rpm) and then washed. We detected OSP-specific antibodies using 100 μL per well of horseradish peroxidase-conjugated goat anti-human IgA at a dilution of 1:1,000 (Jackson Immunoresearch, West Grove, PA) in 0.1% BSA in PBS-0.05% Tween. The plates were incubated at 37°C for 1 hour while shaking (100rpm), washed three times with PBS-0.05% Tween, and developed with ABTS/H_2_O_2_ (Sigma-Aldrich, St. Louis, MO) in 0.1M citrate-phosphate buffer (pH 4.5) and 0.03% hydrogen peroxide. Measurements were made using a kinetic reading at 405 nm wavelength using the SoftMax Pro software.

### Antibody Secreting Cell Responses to OSP and LPS

ASC responses were measured by enzyme-linked immunosorbent spot (ELISPOT) as described previously.[34] To measure the total number of circulating ASCs, nitrocellulose-bottom plates (Millipore, Bedford, MA) were coated with 100μl of affinity-purified goat anti-human IgG F(ab)_2_ (Jackson Immunology Research, West Grove, PA) at a concentration of 5 μg/mL in PBS (pH 7.4). Nitrocellulose plates were also coated with 100μl of the following antigens, to detect *V*. *cholerae-*specific antigen reponses: *V*. *cholerae* O1 Ogawa and Inaba OSP:BSA (10 μg/mL), Ogawa and Inaba-LPS (25 μg/mL), CtxB (2.5 μg/mL;Sigma-Aldrich), and keyhole limpet hemocyanin (KLH, Pierce Biotechnology, Rockford, IL, 2.5 μg/mL). While KLH is a standard control antigen, CtxB was also used to control for ASC responses from possible exposure to natural *V*. *cholerae* O1 infection. The BivWC vaccine lacks recombinant CtxB, therefore should not elicit a detectable CtxB-specific response.

Plates were incubated with antigen overnight at 4°C, and then blocked for 1 hour at 37°C with 200 μL of 2% milk-RPMI. A total of 1 x 10^5^ PBMCs were added to the total Ig-coated wells and serially diluted two times by a factor of 10. A total of 5 x 10^5^ PBMCs were added to the OSP, LPS, CtxB and KLH-coated wells. Plates were then incubated at 37°C for 3 hours and then washed. To detect IgG and IgA ASCs, plates were incubated overnight at 4°C with horseradish peroxidase-conjugated mouse anti-human IgA (Hybridoma Reagent Laboratory, Baltimore, MD) and alkaline phosphatase-conjugated IgG (Southern Biotech, Birmingham, AL), each diluted 1:500 in sterile filtered PBS-0.1%-BSA-tween-0.05%. Following overnight incubation at 4°C, plates were developed with 5-bromo-4-chloro-3-indolylphosphate-nitroblue tetrazolium (BCIP/NBT, Sigma-Aldrich) and 3-amino-9-ethylcarbazole (AEC pre-mix solution, Sigma-Aldrich). Two individuals using a stereomicroscope independently quantified ASC numbers. The number of antigen-specific IgG and IgA ASCs were expressed per 10^6^ total PBMCs and as a percentage of total IgG or IgA expressing cells.

### Statistical Analyses

Statistical analyses were performed using STATA Version 14 (StataCorp, LP, College Station, TX) and Graph-Pad Prism (Graph Pad Software, Inc., La Jolla, CA). Vibriocidal and antigen-specific plasma antibody responses were expressed as geometric mean titers (GMT) with 95% confidence intervals. We used the Wilcoxon signed-rank test to compare within-person antigen-specific ASC responses, vibriocidal antibody responses, and antigen-specific plasma antibody responses across different time points.

We used Spearman’s correlation to quantify associations between antigen-specific ASC, vibriocidal and antigen-specific antibody responses. Comparisons between groups previously exposed and unexposed to *V*. *cholerae* were performed using the Mann Whitney-U test. We tested for equivalence between anti-LPS and anti-OSP ASC responses using two-sided 90% confidence intervals and an equivalence bound of +/- 5 ASCs per 10^6^ PBMCs difference. Confidence intervals within this bound were considered equivalent. All P-values were two-tailed, with a P ≤ 0.05 defined as the threshold for statistical significance.

Our sample size was based on the number of participants needed to detect a difference between the baseline and post-vaccination frequency of LPS-antigen specific ASCs. Extrapolating from other studies,[32,34,39] we estimated that 19 participants would be needed to detect an increase in both IgG and IgA ASC responses, with at least 90% power following vaccination.

## Results

### Study Population

Table 1 describes the demographic characteristics of the 24 individuals that enrolled in the study. One participant dropped out following the first blood draw on day 0, and another dropped out following the second blood draw on day 7. A third participant was excluded from the analysis of ASC responses because of excessive red blood cell contamination in PBMCs at all time points. Thus, 22 participants were included in the analysis of responses following first dose of vaccine; Twenty-one individuals were included in the analysis following the second dose of vaccine. Baseline vibriocidal titers among the study population were consistent with widespread exposure to cholera in a substantial portion of the cohort. 8/22 (36%) participants had a vibriocidal ≥ 80 for the Ogawa serotype, and the same proportion of participants (36%) had a vibriocidal ≥ 80 for the Inaba serotype. A total of 11/22 (50%) had a vibriocidal ≥ 80 for either the Ogawa or Inaba serotype.

**Table 1:** Demographic characteristics of study participants.

Characteristics	Total
	*N = 22*
Mean age, years (S.E.)*	27 (19–50, 10.08)
Gender (%)	
Females	8 (36)
Males	15 (64)
Blood Type (%)	
A	7 (32)
B	4 (18)
O	10 (45)
AB	1 (5)

*S.E. Standard Error

### Plasma Antibody Responses to Vaccination

**Fig 1:**
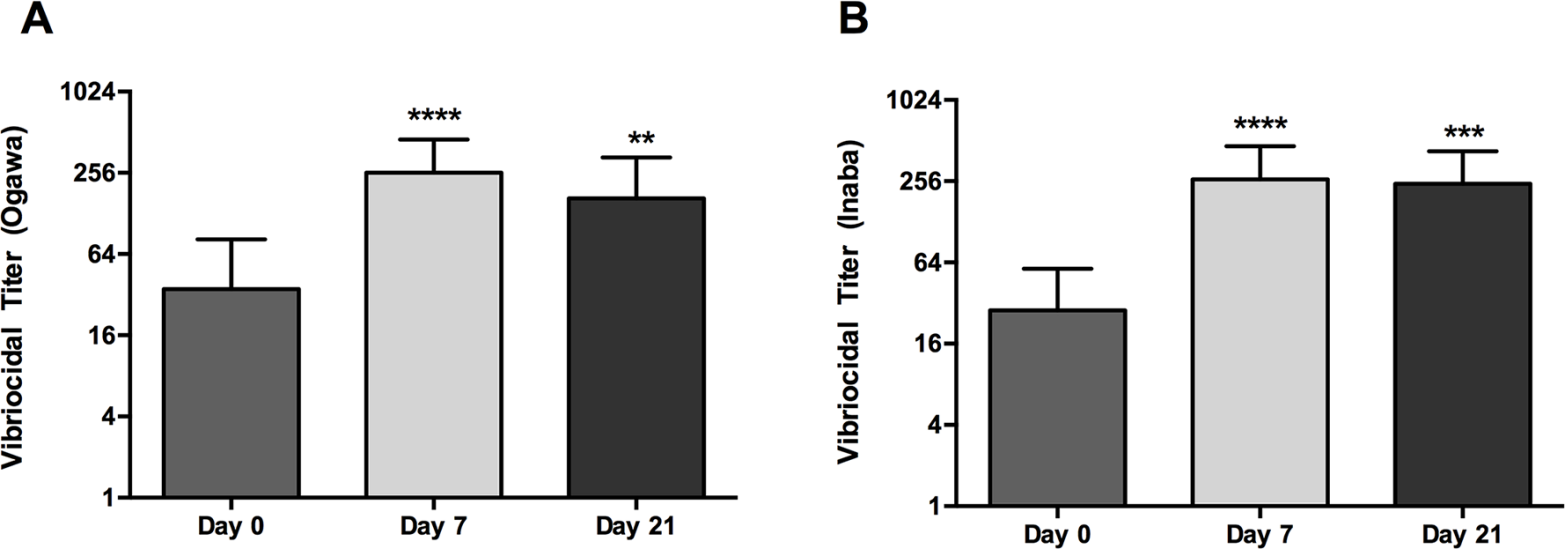
Vibriocidal Responses. Geometric mean titer (+95% CI) of vibriocidal response to *V*. *cholerae* O1 Ogawa (A) and Inaba (B) at baseline (day 0) and 7 days after each immunization (day 7 and 21). Statistically significant differences relative to baseline are indicated (** = P < 0.01, *** = P < 0.001, **** = P < 0.0001).

A summary of immune responses to BivWC at each time point is listed in Table 2. Vibriocidal antibody responses are also depicted in Fig 1. Robust vibriocidal antibody responses to Ogawa and Inaba were detected by day 7 after a single dose of vaccine (p < 0.0001) with a geometric mean fold rise (GMF) of 7.3 to *V*. *cholerae* O1 Ogawa and 9.1 to Inaba. Vibriocidal titers at day 21 were slightly lower compared to day 7 responses, although these differences were not significant. However, vibriciocidal GMT at day 21 remained significantly increased compared to baseline values for Ogawa (p < 0.01) and Inaba (p < 0.001). Vibriocidal antibody seroconversion rates are depicted in Table 2. The majority of participants seroconverted following the first dose of BivWC; Ogawa (64%) and Inaba (73%). The seroconversion rate increased to 76% and 81% for Ogawa and Inaba, respectively, following two doses of vaccine. In addition to vibriocidal antibody responses, we observed significant OSP-specific plasma IgA responses to both Ogawa and Inaba serotypes at days 7 and 21 (Table 2).

**Table 2:** Immune responses to V. cholerae (O1 Ogawa and O1 Inaba) following vaccination with BivWC.

	Total ASC	O1 Ogawa		O1 Inaba	
**Day 0 (*n = 22)***					
Baseline Vibriocidal GMT^a^ *(95% CI)*		35.3	(15.1–82.2)	29.2	(14.7–58.1)
Baseline Serum OSP IgA GMT^b^ *(95% CI)*		65.8	(52.6–82.4)	60.7	(49.6–74.3)
Baseline Total IgA ASCs	334.5 (237.4–431.6)				
Baseline OSP IgA ASCs (per million)		0.02	(-0.02–0.07)	0.07	(-0.01–0.15)
Baseline Total IgG ASCs	920.4 (680.6–1160.1)				
Baseline OSP IgG ASCs (mean, per million)		0.045	(-0.020–0.11)	0.045	(-0.049–0.14)
**Day 7 *(n = 22)***					
Vibriocidal GMT^a^ *(95% CI)*		256.7	(146.0–451.2)	264.9	(150.9–464.9)
Vibriocidal GMF^b^ *(95% CI)*		7.3	(3.4–15.6)	9.1	(4.9–16.9)
Vibriocidal Seroconversion^c^ *(#, %)*		14	64	16	73
Serum OSP IgA GMT^a^ *(95% CI)*		93.6	(66.1–132.7)	86.6	(64.0–117.3)
Serum OSP IgA GMF^b^ *(95% CI)*		1.4	(1.0–1.9)	1.4	(1.1–1.9)
Serum OSP IgA Seroconversion^d^ *(#, %)*		7	32	4	18
Total IgA ASCs	290 (233.5–346.5)				
OSP IgA ASCs (mean, per million)		6.9	(1.9–12.0)		
OSP IgA ASCs Responders^e^ *(#, %)*		13	59		
Total IgG ASCs	1017 (666.7–1367.3)				
OSP IgG ASCs (mean, per million)		1.2	(0.58–1.87)	0.36	(-0.0059–0.73)
OSP IgG ASCs Responders^e^ *(#, %)*		5	22.7	3	13.6
**Day 21 *(n = 21)***					
Vibriocidal GMT^a^ *(95% CI)*		165.4	(81.0–333.6)	245.7	(141.7–426.3)
Vibriocidal GMF^b^ *(95% CI)*		4.9	(2.5–9.6)	9.1	(4.6–18.2)
Vibriocidal Seroconversion^c^ *(#, %)*		16	76	17	81
Serum OSP IgA GMT^a^ *(95% CI)*		86.4	(66.0–113.1)	83.5	(65.6–106.5)
Serum OSP IgA GMF^b^ *(95% CI)*		1.4	(1.1–1.9)	1.5	(1.2–1.8)
Serum OSP IgA Seroconversion^d^ *(#, %)*		8	38	6	29
Total IgA ASCs	408.3 (306.9–509.7)				
OSP IgA ASCs (mean, per million)		1	(0.1–1.9)	0.8	(0.1–1.5)
OSP IgA ASCs Responders^e^ *(#, %)*		5	23.8	5	23.8
Total IgG ASCs	1035.1 (786.3–1283.9)				
OSP IgG ASCs (mean, per million)		0.41	(-0.083–0.90)	0.2	(-0.091–0.50)
OSP IgG ASCs Responders^e^ *(#, %)*		1	4.8	1	4.8

^a^ Geometric mean titer.

^b^ Geometric mean-fold rise from baseline to 7 days after 1^st^ dose or from baseline to 7 days after 2^nd^ dose.

^c^ # with ≥ 4-fold rise in titers from baseline to 7 days after 1^st^ dose or from baseline to 7 days after 2^nd^ dose.

^d^ 3 with ≥ 2-fold rise in titers from baseline to 7 days after 1^st^ dose or from baseline to 7 days after 2^nd^ dose.

^e^ # with ≥ 2 ASCs detected

^#^ Number of participants

### Antibody Secreting Cell (ASC) Responses to Vaccination

We did not observe an overall increase in the number of total IgA and IgG antibody secreting cells either in total or relative to the total number of peripheral blood mononuclear cells following vaccination with BivWC (Table 2). Among antigen-specific responses, there was no response to either of the control antigens, CtxB or KLH, at any time point. Some artefactual spots (typically <3) are typical of ELISPOT assays. We observed a single KLH spot in only three of the 130 ELIPSPOT samples analyzed (2.3%), and no more than a single KLH spot was observed in any of the samples. The low number of KLH spots suggests that there was negligible artefactual background in the other antigen-specific ASC measurements. Similarly, in four of the 130 samples (3.1%) analyzed as part of the study, we observed a single CtxB spot, and again, in no samples, were more than a single CtxB spot observed. As such, we defined the presence of ≥ 2 ASCs per 10^6^ PBMCs as a positive ASC response. The lack of CtxB responses suggests that responses detected to the OSP or LPS antigens were not likely the result of intercurrent infection with or exposure to *V*. *cholerae*, since natural infection is associated with significant responses to the CtxB antigen.

**Fig 2:**
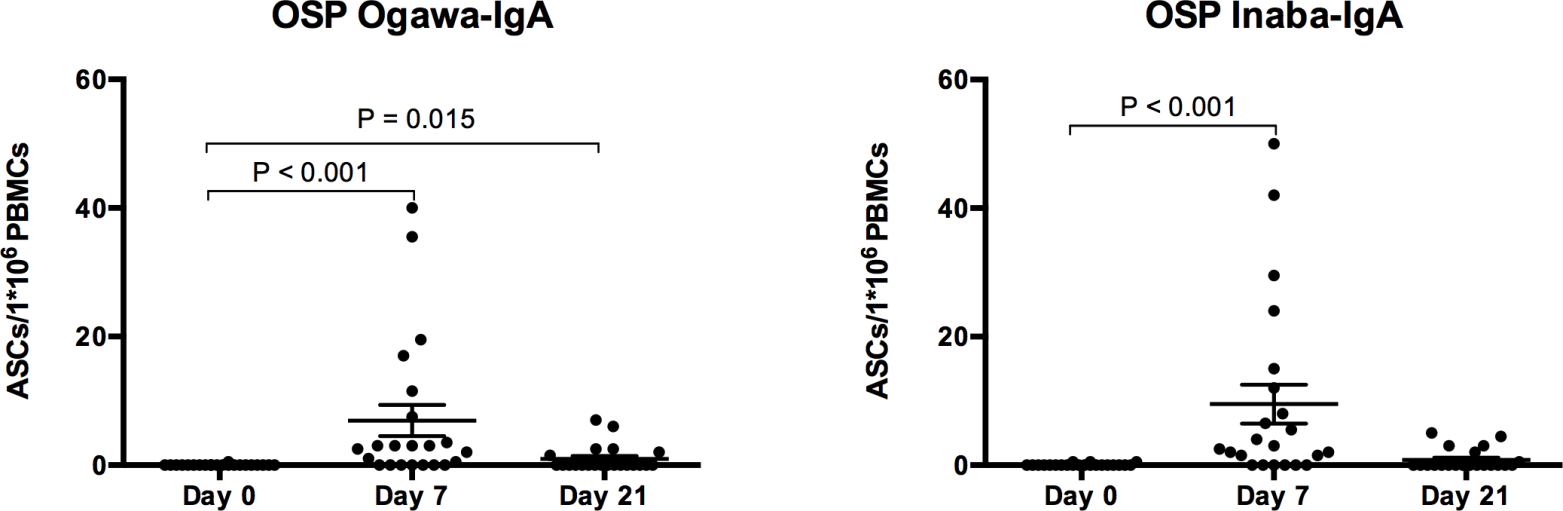
Ogawa and Inaba O-specific polysaccharide (OSP) Immunoglobulin A (IgA) Antibody secreting cell (ASC) responses in vaccinees. Individual and mean circulating antigen-specific IgA responses to Ogawa and Inaba OSP with standard error bars. P values are indicated where statistically significant differences from baseline (Day 0) are present.

OSP-specific ASC responses are shown in Fig 2. Notably, OSP-specific IgA ASC responses to Ogawa and Inaba peaked on day 7 following the first dose of vaccine, and were significantly increased from baseline (P < 0.001). Notably, day 7 OSP-specific IgA antibody responses detected in plasma were strongly correlated with day 7 OSP-specific IgA ASC responses to Ogawa (Spearman r = 0.6877, P = 0.0004) and Inaba (Spearman r = 0.6077, P = 0.0027)

An analysis of ASC response based on stratification by a pre-vaccination vibriocidal titer of ≥ 80, shows no significant difference in mean ASC responses. OSP-specific IgA responses at day 7 to the Ogawa serotype were 5.2 ASCs per million PBMCs and 7.9 ASCs per million PBMCs among those exposed and unexposed (P = 0.8894), respectively, whereas OSP-specific IgA responses to the Inaba serotype were 10.4 ASCs per million PBMCs and 9 ASCs per million PBMCs (P = 0.285), respectively. Additionally, no significant differences were seen when comparing OSP-specific IgA ASC responses at day 7 to the Ogawa and Inaba serotype between individuals with previous exposure to either Ogawa or Inaba (P = 0.6905 for Inaba OSP response, P = 0.7889 for Ogawa response). However, our sample size is limited and not sufficiently powered to definitively assess whether prior exposure in this setting impacts mucosal immune responses.

**Fig 3:**
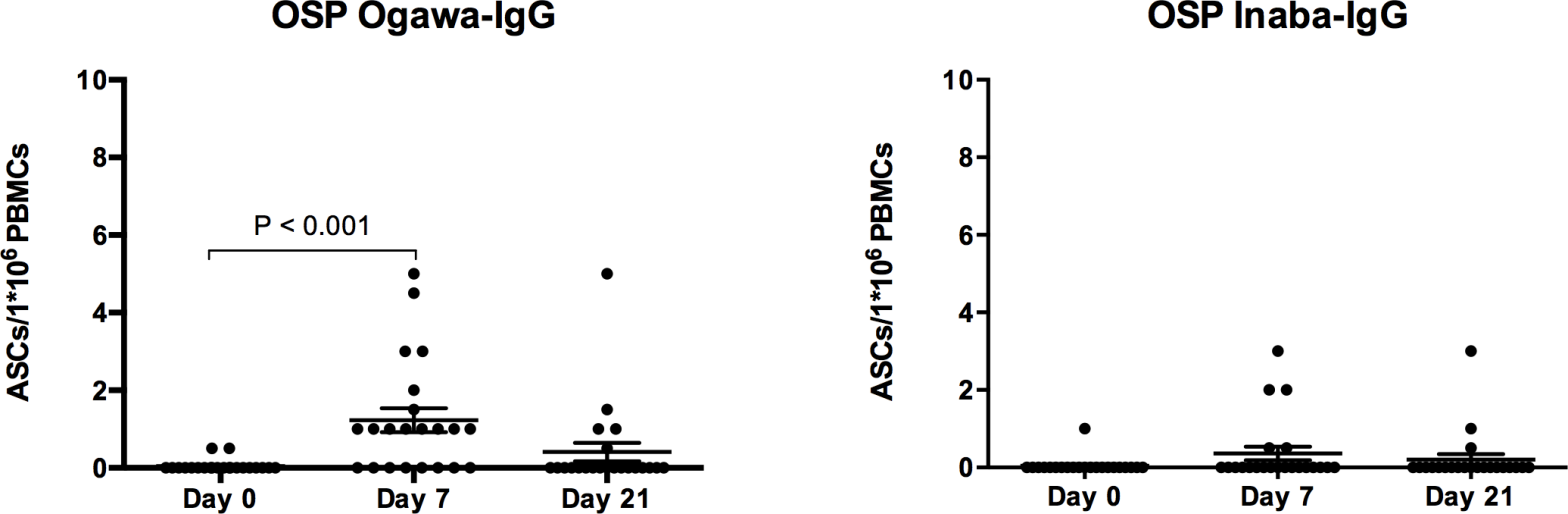
Ogawa and Inaba O-specific polysaccharide (OSP) Immunoglobulin G (IgG) Antibody secreting cell (ASC) responses in vaccinees. Individual and mean circulating antigen-specific IgG responses to Ogawa and Inaba OSP with standard error bars. P values are indicated where statistically significant differences from baseline (Day 0) are present.

OSP-specific IgA ASC responses after the second dose of the vaccine decreased nearly to baseline levels by day 21. OSP-specific IgG antibody responses were proportionally lower than IgA responses, although OSP-specific IgG ASC responses nonetheless demonstrated a statistically significant increase from baseline for the Ogawa (p < 0.001), but not the Inaba serotype (p = 0.099) (Fig 3).

**Fig 4:**
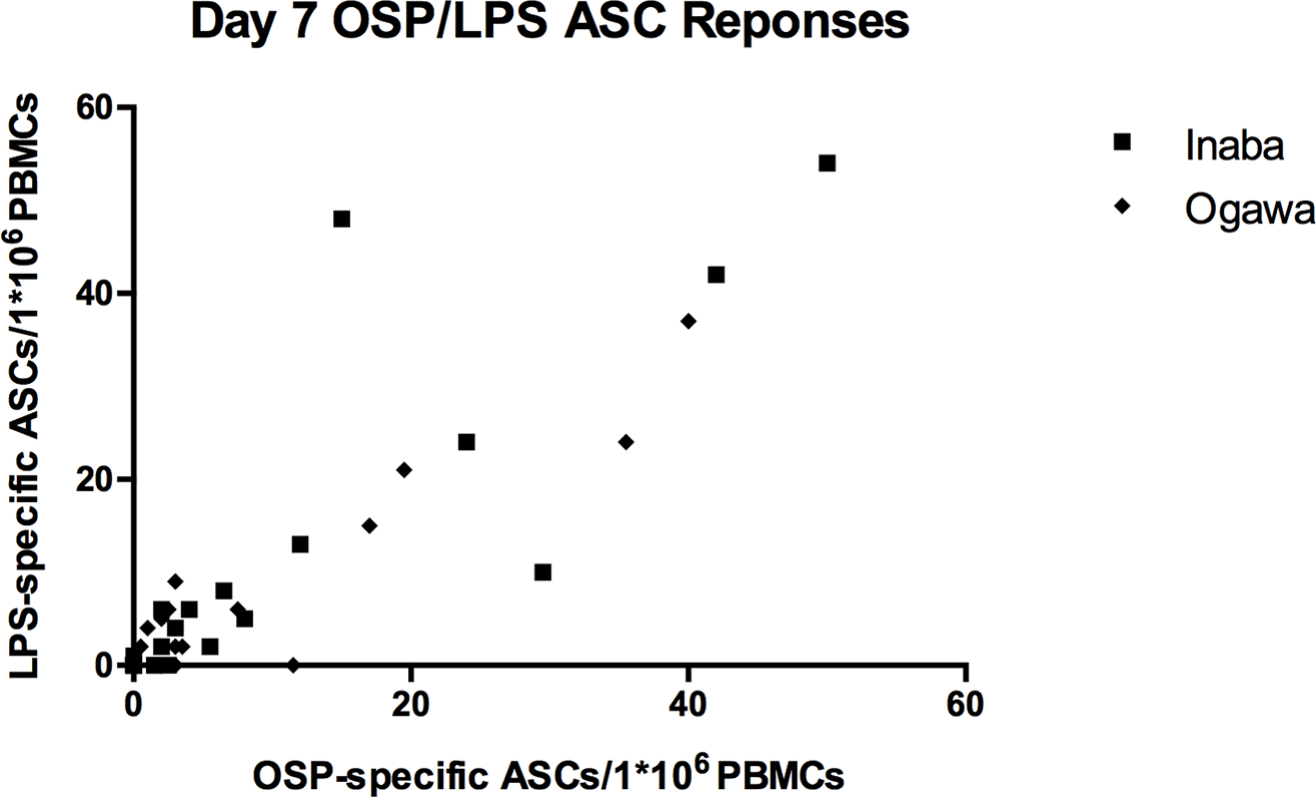
Correlation between Day 7 anti-OSP and anti-LPS ASC Responses. Ogawa: Spearman’s r = 0.7706, p < 0.0001. Inaba: Spearman’s r = 0.9030, p < 0.0001.

We noted the OSP and LPS responses were not only highly correlated, but appeared nearly equivalent. (Fig 4) Testing for equivalence of IgA ASC responses to LPS and OSP demonstrated a mean difference of 0.70 ASCs per million PBMCs (90% CI: -0.799–2.21) and 0.73 ASCs per 10^6^ PBMCs (90% CI: -3.86–2.41) for Ogawa and Inaba, respectively. The confidence intervals for these mean cell differences lie within the predetermined equivalence range of +/- 5 ASCs per 10^6^ PBMCs, suggesting equivalence or near equivalence between anti-LPS and anti-OSP ASC responses at a significance level of 0.05.

## Discussion

To our knowledge, this study is the first report of ASC responses to the BivWC oral cholera vaccine. We demonstrate that when administered according to the recommended two dose schedule in adults living in Haiti, BivWC induces a robust mucosal response to the *V*. *cholerae* O1 antigen following the first vaccine dose. However, a second dose of the vaccine administered 14 days later did not result in a robust mucosal response to the *V*. *cholerae* O1 antigen.

*V*. *cholerae* is a non-invasive pathogen, and it is thought that immunity against cholera is most likely mediated by *V*. *cholerae* O1-antigen specific IgA produced at the mucosal surface. For this reason, IgA-secreting ASCs, which transiently appear in the circulating following vaccination and ultimately differentiate into mucosal plasma cells provide an important window into mucosal immunity. In this context, our finding that a second dose of the OCV administered 14 days after the first dose does not result in significant ASC responses in Haitian adults raises important questions about the appropriate dosing interval for the BivWC vaccine in cholera-endemic areas. While the 14-day interval may allow for the delivery of two vaccine doses to people during an outbreak as quickly as possible, it is not clear whether this is a sufficient time period to generate sufficient immunologic memory in order to achieve a prime-boost effect with the second vaccine dose. Given that our findings mirror previous studies conducted in cholera-endemic areas of South Asia following vaccination with the WC-rBS vaccine,[32,34,40] we suspect that the current 14-day dosing schedule is too short for the generation of memory B cells that can respond to secondary antigenic stimulation upon re-exposure to *V*. *cholerae* O1 OSP.

To address this question regarding the timing of the second vaccine dose definitively would require additional studies. Specifically, evaluating the effect of re-vaccination with oral cholera vaccine at different intervals and determining whether repeated vaccination results in increasing levels of class switching and affinity maturation among the resulting *V*. *cholerae*-specific ASCs, would determine whether *bona fide* functional and long lasting B cell memory can be boosted following repeated doses of oral cholera vaccination administered at longer intervals. These results could then provide mechanistic context for larger vaccine effectiveness studies comparing both single and multi-dose OCV regimens.

Another notable finding in this report was derived from measuring ASC responses separately to both *V*. *cholerae* O1 LPS and the O-antigen-specific polysaccharide (OSP) moiety of LPS. These responses were not only strongly correlated, but also nearly equivalent, suggesting that immune responses primarily target the OSP portion of the antigen, rather than the O-antigen core, lipid A, or other component of the LPS preparation. These findings have implications both for subsequent evaluations of immune responses following cholera infection or vaccination, and the development of more immunogenic vaccines for cholera.

Another finding in our study, was the significant correlation we observed between day 7 antigen-specific plasma IgA responses and day 7 antigen-specific ASC responses. This correlation has been previously described following infection with *V*. *cholerae* or vaccination, [8,34] and suggests that plasma IgA responses on day 7 following vaccination does indeed serve as a potential proxy for the mucosal ASC response in some circumstances.

Our study has a number of limitations. Because cholera is now endemic in Haiti, our cohort had high pre-existing levels of vibriocidal antibodies before vaccination with 50% of participants having a vibriocidal ≥ 80 for either the Ogawa or Inaba. These findings are in line with a serosurvey conducted in Haiti in 2011 demonstrating that 64% of individuals had a vibriocidal antibody titer ≥ 80 for either *V*. *cholerae* Inaba or Ogawa, suggesting high levels of prior infection.[41] This is important because ASC responses to vaccination are likely to differ between populations based on the level of previous antigen exposure. For example, in contrast to our results in Haiti where evidence of cholera exposure was common, a previous study conducted among Swedish volunteers demonstrated significant CT-specific ASC responses to a WC-rBS vaccine following the second dose of vaccine as part of a similar vaccination schedule spaced 14 days apart, suggesting differences between responses in endemic and non-endemic settings.[39] Levels of previous exposure also effect the kinetics of the ASC response, and ASC responses peak earlier among primed populations.[42] Because we only measured ASC responses at a single time point after each vaccination (7-days) we could not evaluate the kinetic aspects of the response. While this is a limitation of the study, we do not think it changes the major findings, because based on previous studies, it is very unlikely that such priming would shift the kinetics of ASC responses to the extent they would become undetectable by day 7.[42] Lastly, while our results should be generalizable to adults living in other cholera endemic areas, additional studies are also needed in children and other subpopulations in cholera-affected regions, who may differ in their mucosal response to the BivWC vaccine.

In conclusion, vaccination with the BivWC oral cholera vaccine induces a significant mucosal ASC response targeting the OSP portion of *V*. *cholerae* LPS in Haitian adults, while a second dose of vaccine does not appear to stimulate antibody secreting cell responses in this population. These findings are similar to responses with the WC-rBS oral cholera vaccine in other cholera-endemic regions. More detailed evaluations of whether alternate vaccine dosing intervals can stimulate more robust mucosal immune responses, and contribute to improved duration of memory B cell targeting the *V*. *cholerae* O-antigen should be a priority for future studies.

## Supporting Information

S1 Dataset Table containing all relevant study data. (XLS)
